# A Predictive and Preventive Model for Onset of Alzheimer's Disease

**DOI:** 10.3389/fpubh.2021.751536

**Published:** 2021-10-11

**Authors:** Udit Singhania, Balakrushna Tripathy, Mohammad Kamrul Hasan, Noble C. Anumbe, Dabiah Alboaneen, Fatima Rayan Awad Ahmed, Thowiba E. Ahmed, Manasik M. Mohamed Nour

**Affiliations:** ^1^School of Computer Science and Engineering, Vellore Institute of Technology, Vellore, India; ^2^School of Information Technology and Engineering, Vellore Institute of Technology, Vellore, India; ^3^Center for Cyber Security, Faculty of Information Science and Technology, Universiti Kebangsaan Malaysia (UKM), Bangi, Malaysia; ^4^Department of Mechanical Engineering, University of South Carolina, Columbia, SC, United States; ^5^Computer Science Department, College of Science and Humanities, Imam Abdulrahman Bin Faisal University, Jubail, Saudi Arabia; ^6^Computer Science Department, College of Computer Engineering and Science, Prince Sattam Bin Abdulaziz University, Al-Kharj, Saudi Arabia; ^7^Department of Mathematics, College of Science and Humanity, Prince Sattam Bin Abdulaziz University, Al-Kharj, Saudi Arabia

**Keywords:** machine learning, Alzheimer's disease, prediction, prevention, convolutional neural networks, support vector machine

## Abstract

Alzheimer's Disease (AD) is a neurodegenerative irreversible brain disorder that gradually wipes out the memory, thinking skills and eventually the ability to carry out day-to-day tasks. The amount of AD patients is rapidly increasing due to several lifestyle changes that affect biological functions. Detection of AD at its early stages helps in the treatment of patients. In this paper, a predictive and preventive model that uses biomarkers such as the amyloid-beta protein is proposed to detect, predict, and prevent AD onset. A Convolution Neural Network (CNN) based model is developed to predict AD at its early stages. The results obtained proved that the proposed model outperforms the traditional Machine Learning (ML) algorithms such as Logistic Regression, Support Vector Machine, Decision Tree Classifier, and K Nearest Neighbor algorithms.

## Introduction

Alzheimer's disease (AD) is 60–70% the cause of dementia (1, 2). It is a slowly progressing brain disorder. The individuals that express symptoms of AD have abnormal deposition of a protein called amyloid-beta in their brain. This amyloid-beta protein forms plaques in the brain and strands of protein tau twists around, causing tangles that ultimately kills the brain cells. The degeneration of brain cells causes loss of memory, thinking and reasoning skills (3).

A few investigations have demonstrated that trained radiologists can be outflanked by computer-helped-diagnosis utilizing Support Vector Machines (SVMs) in distinguishing patients with AD and different ailments (4). Convolutional Neural Networks (CNNs) are simply neural networks that use convolution in place of general matrix multiplication in at least one of their layers (5). These are special type of Deep Neural Networks (DNNs) (6). Several applications of DNN can be found in (7). Deep learning techniques are used abundantly used in healthcare (8). For example various uses of CNNs for Magnetic Resonance Imaging (MRI) segmentation are presented in (9). CNNs are used for several applications Different groups have tried utilizing CNNs (10, 11) to analyze and separate AD from healthy or no condition (NC), patients using MRI scans as an input, while others have utilized a special type of MRI scan technique—the functional magnetic resonance imaging (fMRI) time-series information (12, 13). An investigation was performed by Thompson et al. (14) in this regard in a paper titled, “Applying Convolutional Neural Networks for pre-detection of AD from structural MRI data.” In this study, the authors utilized SVMs and CNNs on sectioned areas of interest after post-processing utilizing edge-detection algorithms on grouped MRI scan. This study achieved a sensitivity of 96% and a specificity of 98% on a dataset of 1,615 MRI images. Segmented images with edge-detection were used to classify them according to the required to the required quality standards.

The level of amyloid protein in the brain varies from individual to individual. Researchers have found (15, 16) that the rate of death from Alzheimer's has increased by 50% in the recent years, from 16 deaths in 1,00,000 in 1999 to 25 deaths in 1,00,000 in 2014. Also, the number of people who have died of AD has increased two-fold, from 44,536 in 1999 to 93,541 in 2014. In 2015, there were about 29.8 million people worldwide affected by AD. The cause of AD is still not properly understood. About 70% of the cause is inferred to be genetic in nature (17). A methodology to track, predict and cure AD can be analyzed with the history of the illness, cognitive testing with medical imaging and blood tests. People affected by AD chiefly rely on others for assistance which often turns out to be a burden on the caretakers. This results in social, psychological, physical, and economic pressure (18). Hence, we propose a model that would accept Positron Emission Tomography (PET) brain scans as inputs, process and analyze them and finally predict whether the individual will have Alzheimer's within a given time window of two years. It is said that prevention is better than cure. Hence, early detection of the onset of AD is highly beneficial. The techniques that are currently available to detect AD rely on cognitive impairment testing which is not efficient in yielding accurate diagnosis. The cognitive tests mostly rely on results of questionnaires directed toward the subjects and do not consider medical developments within the subject's brain explicitly. Such tests will prove to be an assuring method to predict the level of onset of AD in a subject only if there exists a one-on-one correspondence between the results of the tests and the physical state of the brain (the concentration of proteins that regulate the onset of AD). Thus, a new technique or algorithm was proposed in (19) with the aim of reducing the high dimensional MRI vector space to 150 dimensions using Principal Component Analysis (PCA). To categorize the reduced dimensions to PCA for progression of AD, multi-class neurons were employed. In comparative studies as in (20), Percent Whole Brain Volume Change (PBVC) was measured from serial MRI scans and dementia with Lewy bodies. The conclusion was that the atrophy of AD was significantly greater than that of Dementia with Lewy bodies (DLB) for one year in various regions of the brain including periventricular areas. PBVC was not significantly different from DLB and it was concluded that AD showed faster rate of global growth than DLB. Certain research has also been done to determine alterations occurring in Parahippocampal Cigumul bundle (PhC) and Posterior Cingulum bundle (PoC) in patients suffering from Mild Cognitive Impairment (MCI) (21, 22). This was done through diffusion tensor imaging (23–28). An atlas-based Region-Of-Interest (ROI) was used to calculate the fractional anisotropy, mean diffusivity, axial diffusivity, and radial diffusivity. For the primary health centre (PhC), a significant decrease was observed in the FA value, whereas an increase was observed in the MD and RD values. It is forecasted that by 2050, the prevalence of AD will quadruple to 26.6 million cases, where approximately 43% of them will need a high level of care. If the diagnosis of AD can be improved upon and treated at an earlier and more manageable stage, where therapeutics and preventive care are more useful, then the numbers of future AD patients will likely decrease.

The aim of this paper is to bring forward a predictive model for early prediction of AD and thus, develop a preventive model based on it. This model would be helpful in identifying patients with AD and those at risk of developing it. Furthermore, we suggest a preventive course of action to be followed to limit the growth in AD. In this paper, we propose an integrated algorithm that identifies a patient with AD and predicts the onset of AD with a data-oriented approach. In the predictive model, we consider amyloid protein concentration in MRI scans as one of the biomarkers to predict the onset of AD within a window of 2 years. Initially, based on MRI scans and cross sectional and longitudinal MRI scan datasets, we classify subjects as AD or No Condition (NC). Following this, we analyze the datasets to compute the accuracy of onset of AD for the subjects. This is without considering the subjects classified as AD or NC by the first algorithm. We further use this to outline the preventive measures that should be taken to prevent (or delay) the onset or worsening of AD. The preventive measures are aimed at reducing the Critical Design Reviews (CDR) score and hence, reduce the severity of the onset of AD. The rest of the paper is organized as follows: In materials and methods, we broadly discuss the algorithm that we suggest with the data used, the model description and the performance of the algorithm. In results and analysis, we analyze the results of our algorithm and discuss the same. In result analysis, we provided a conclusion that briefly describes results and analysis and provides further improvement and upgrades that can be made in the proposed algorithm.

## Materials and Methods

In this section, we broadly discuss the architectural design of the algorithm, the types of data used and the methods of data acquisition. We also discuss the performance of the algorithm and the classification algorithms used.

### Subjects and Data Acquisition

We have taken a data-oriented approach while developing the algorithm. This algorithm is developed consulting the existing algorithms. The data used for the algorithm was obtained from an Operational Applications of Special Intelligence Systems (29–32). It consists of cross-sectional MRI scan collection of 416 individuals within the age group of 18 to 96. About 100 of the individuals mentioned in the dataset were clinically diagnosed with mild or moderate AD. The data used here also comprises of longitudinal MRI scan collection of 373 individuals within the age group of 18 to 96. The dataset consisted of biomarkers that have been scientifically proven to predict onset of AD. The primary attributes of the dataset were the Minimal Mental State Examination (MMSE) scores and the Clinical Dementia Rating (CDR) scores.

### Model Description

**Figure 1:**
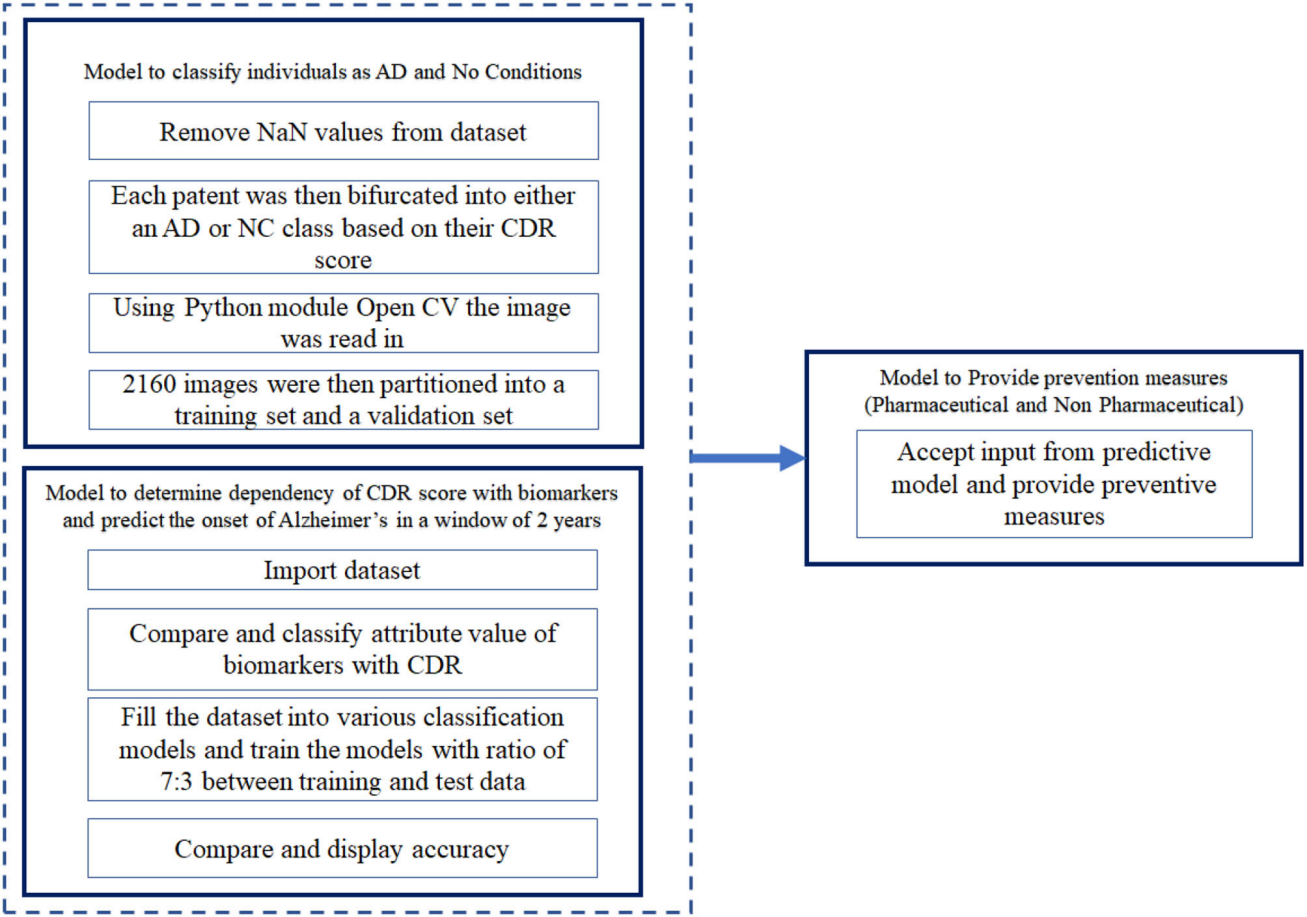
Architecture of the system.

The algorithm developed can be visualized as two modules, namely, the predictive model and the preventive model. The predictive model takes into consideration the dataset mentioned above and uses it to predict the possibility of onset of AD (Figure 1). The predictive model is divided into two sub models. The first model of the predictive algorithm takes into consideration the cross sectional dataset and the MRI scan images of the subjects mentioned in the cross sectional dataset and confirms whether a given subject is affected by AD currently. Initially, it removes null values from the dataset by dropping rows that have one such value. Due to unavailability of diverse range of Critical Design Reviews (CDR) scores, any individual having a CDR score equal to 0 was considered to be Non-Cognizable (NC) and the remaining CDR scores implied AD. Now, the removal of rows consisting of “NaN” values reduced the datasets to 216 data points and each of the individuals had 10 coronal slices of MRI scans.

Using the OpenCV algorithm available, the images were read as a numpy array. This was then normalized by dividing each intensity by 255, which is the maximum intensity. The k-means algorithm was run on the pixel space of each image. In the analysis phase, only two clusters were chosen for simplicity and maximum noise reduction. Each pixel was then assigned to the cluster it was in. The 2,160 image set obtained was divided into training set and validation set, such that a ratio of 7:3 was maintained between the training and validation set. The training set was then fed into the CNN, VGG16 in batches of 32. The model flattens the output and feeds it into two fully-connected or dense layers each containing 1,028 connecting units. Using a softmax activation function in the final layer, stochastic gradient descent as an optimizer, categorical cross entropy as a loss, the model was trained for 150 epochs. This algorithm classifies individuals with present traits of AD. In the second model of the predictive algorithm, the cross sectional and longitudinal datasets were fit into various classification models such as Decision Tree Classifier, Logistic Regression, TensorFlow, K nearest neighbour (KNN) (33–35), SVM etc. and compare the accuracy with which these classification algorithms predict the CDR scores when provided the set of biomarkers and parameters available in the dataset. In the preventive model, we consider the most recent CDR score of the subject and accordingly provide the clinically approved preventive measure.

### Performance Measurement and Classification

For a fixed set of Access Points (Aps), the probability of a user to be closer to any three out of the four APs is greater than that of being at equal distances from each of the APs. At every user location, the updated list of three APs based on maximum RSSI and minimum Program Visualization (PV) distances are utilized in the *k*-NN search to get the least localization error. Maximization of the RSSI objective function is computed as;


(1) $$\begin{array}{l} f\left(x\right)=B\left(0\right)+\sum_{i=1}^{n}\left(a_{i}*\left(x,x_{i}\right)\right) \end{array}$$

Here, *x* represents the new input vector and *a*_*i*_*, x*_*i*_ represents all the weights of the neural and the support vectors obtained from the training data respectively. *B (0)* is the bias input chosen. On the other hand, logistic regression is used for the binary classification and prediction using the parameters and biomarkers mentioned above to classify the subjects as AD or NC based on the MMSE and CDR scores available in the training data. The logistic function used to obtain the predicted value is given by:


(2) $$\begin{array}{l} f\left(x\right)=B\left(0\right)+\sum_{i=1}^{n}e^{\left(b_{0}+b_{i}*x\right)}/\left(1+e^{\left(b_{0}+b_{i}*x\right)}\right) \end{array}$$

Where *b*_0_ represents the bias used in the network and *b*_*i*_ represents the vector values obtained from the training data. There were other classification algorithms used to predict the CDR scores. The architecture of the proposed system is depicted in Figure 1.

## Results and Analysis

**Figure 2:**
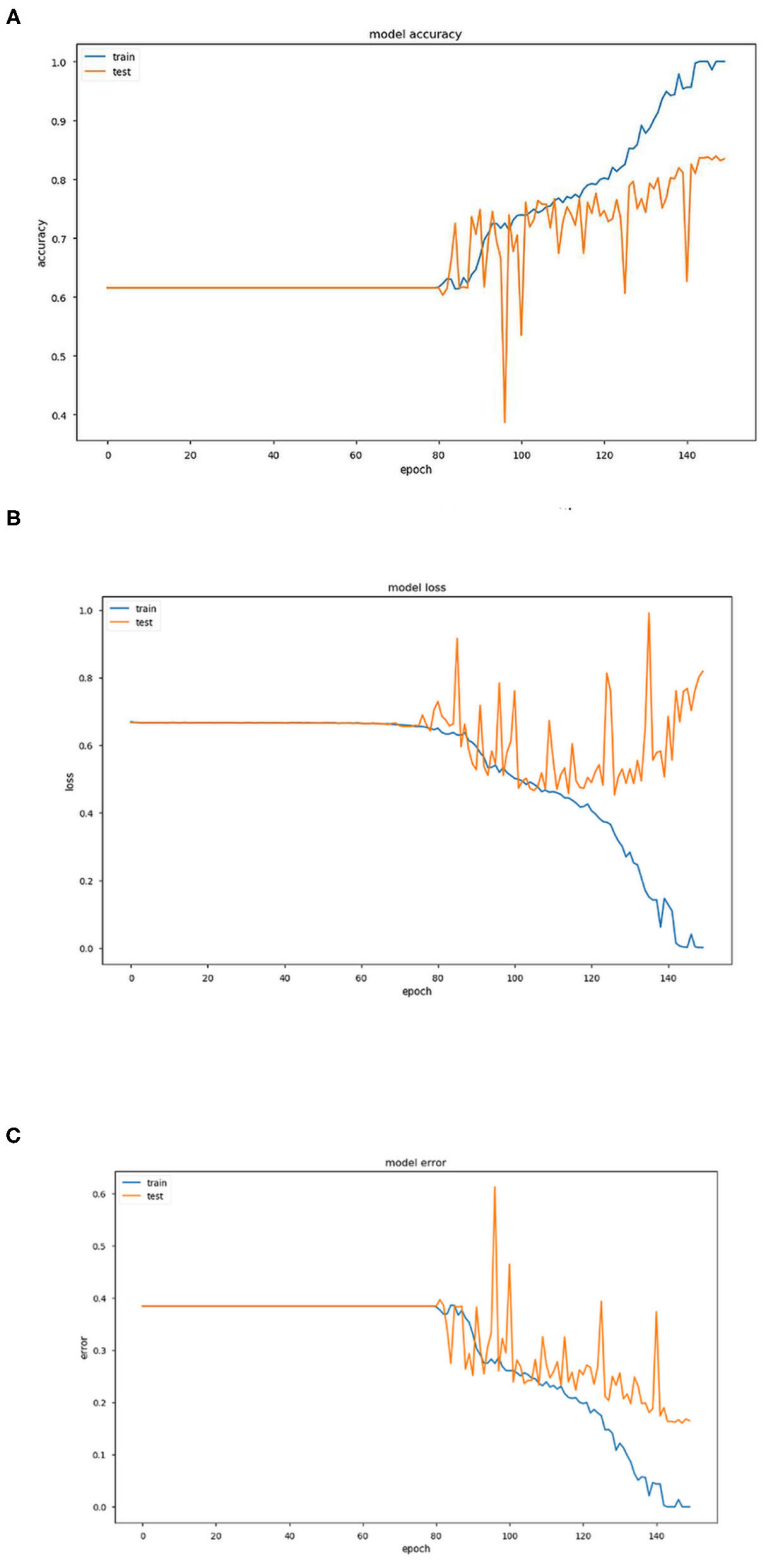
**(A)** Graph representing variation of model accuracy with respect to epoch. **(B)** Graph representing variation of model loss with respect to epoch. **(C)** Graph representing variation of model error with respect to epoch.

**Figure 3:**
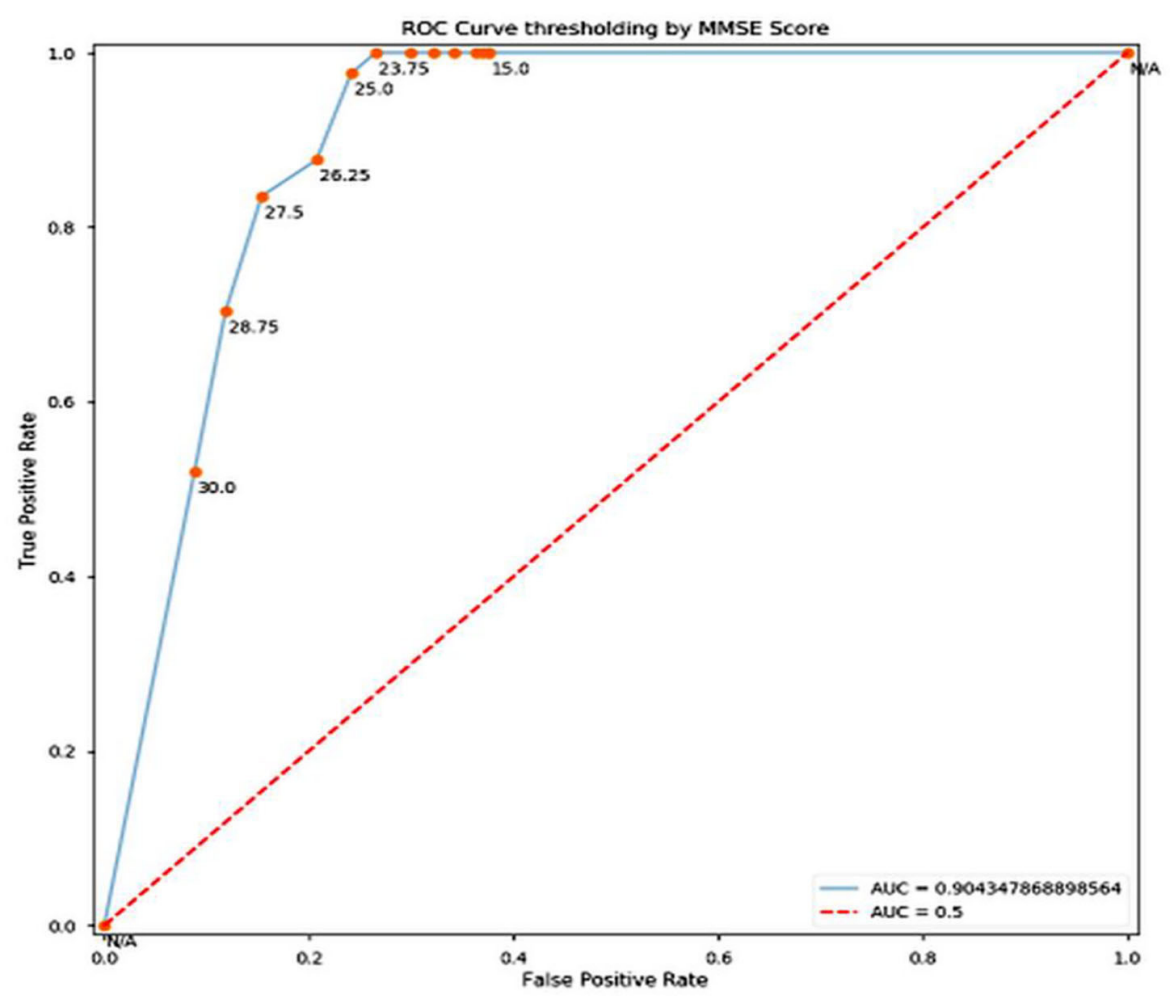
ROC Curve.

In this section, we mention the results of the algorithm and discuss the same. The experimental evaluation approach has considered for the assessment of the proposed method. The algorithms were run on Intel(R) Core (TM) i5-7200U CPU with 2.50GHz. The experimental result is presented in Figures 2, 3.

The above three Figures 2A–C provide estimations of the model accuracy, model loss and model error with respect to the increase in epoch during the training phase and testing phase. Before epoch reaches 80, all the three parameters of accuracy, loss and error remain constant and also coincide. The variations occur after this in the training phase and the accuracy increases continuously, the loss and error decrease as expected. In the testing phase also, the same trend is observed but the extent becomes much restricted with large variations.

Training and test accuracy were measured at each epoch. The model stagnated and predicted that all brain scans were NC for the first 81 epochs. Then it moved out of its local minima and began increasing its accuracy. The training error ultimately became 0% at epoch 144, thus showing it had predicted all the brains correctly. This can be taken as an indication of overfitting on the training set. However, the lowest prediction error reached is 16.05%, by epoch 148. It can be argued that there could be a further decrease in the prediction error. But due to the model reaching 0% training error, it was stopped prematurely. As a consequence a part of the training dataset is typically set aside as the “test set” to check for overfitting.

### Predictive Model

For the algorithm that predicts whether a person has AD or not, each epoch took around 1,890 s to train. The total number of epochs run was 150, which took about 4,725 min or around 79 h.

Training and test accuracy were measured at each epoch. The model stagnated and predicted that all brain scans were NC for the first 81 epochs. Then it moved out of its local minima and began increasing its accuracy. The training error ultimately became 0% at epoch 144, thus showing it had predicted all the brains correctly. This can be taken as an indication of overfitting on the training set. However, the lowest prediction error reached is 16.05%, by epoch 148. It can be argued that there could be a further decrease in the prediction error. But due to the model reaching 0% training error, it was stopped prematurely An ROC (Receiver Operating Characteristic) Curve was plotted by thresholding against a patient's Minimum Mean-Square Error (MMSE) scores, to evaluate the model (Figure 3). The ROC is a graphical plot that illustrates the diagnostic ability of a binary classifier system as its discrimination threshold is varied.

In conclusion, it can be stated that this model of pre-processing with k-means and training with VGG16 performed well (training accuracy = 100%, prediction accuracy = 83.95%, AUC = 0.904). It can perform better by increasing the number of clusters in the k-means step, combining edge-detection and segmentation steps, using better hardware and larger networks in terms of the number of layers and epochs run, and in general obtaining of more data. In terms of engineering the biological system of the brain in the context of AD, this model does well in interpreting the brain as a set of pixels and their intensities but easily disregards the numerous other variables that assemble a diagnosis, especially considering that MRI scans are already a large abstraction from the vastly complex human brain.

**Figure 4:**
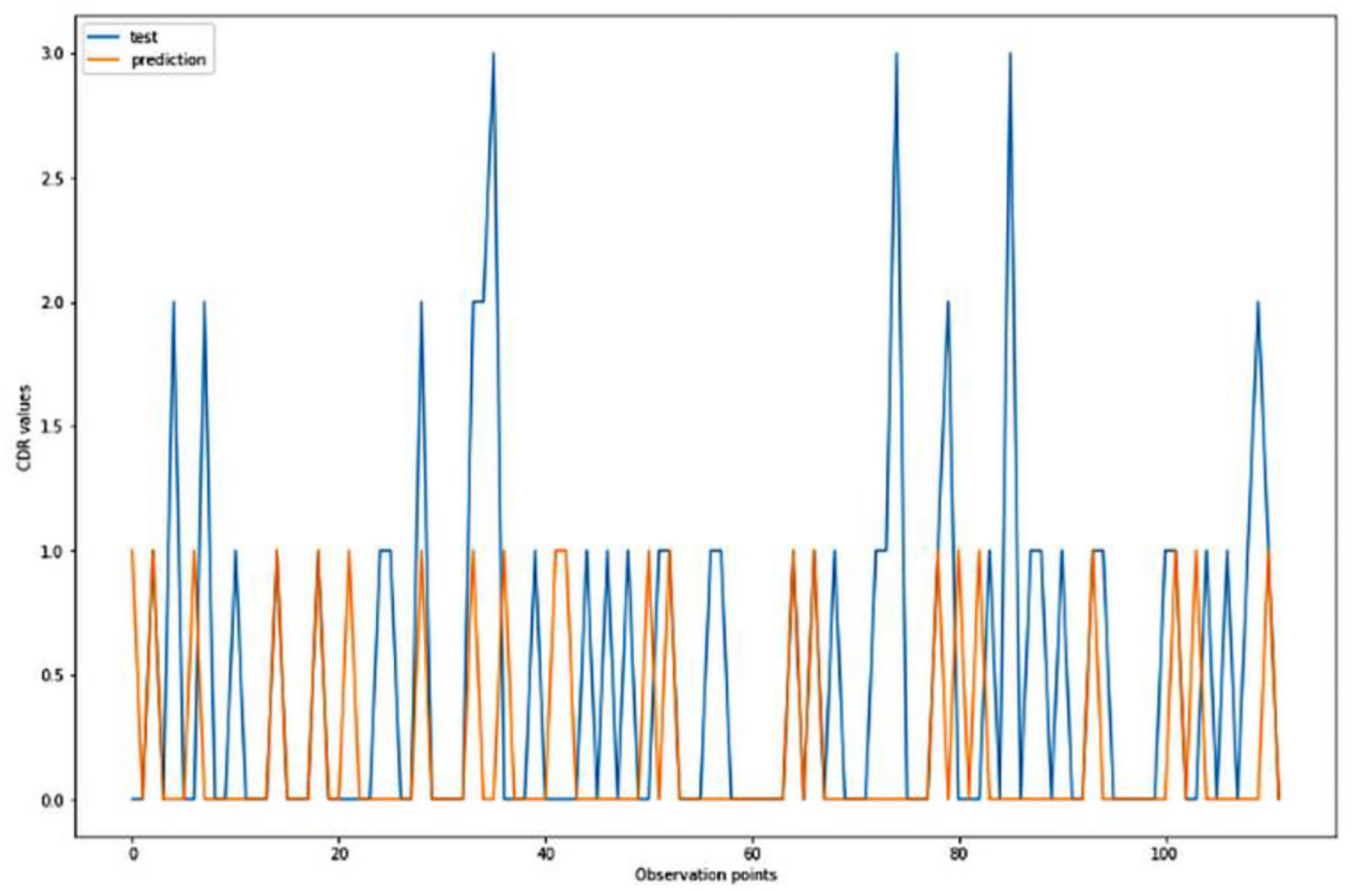
Graphical representation of the comparison of test and predicted CDR values obtained using the logistic regression algorithm. (six parameters).

**Figure 5:**
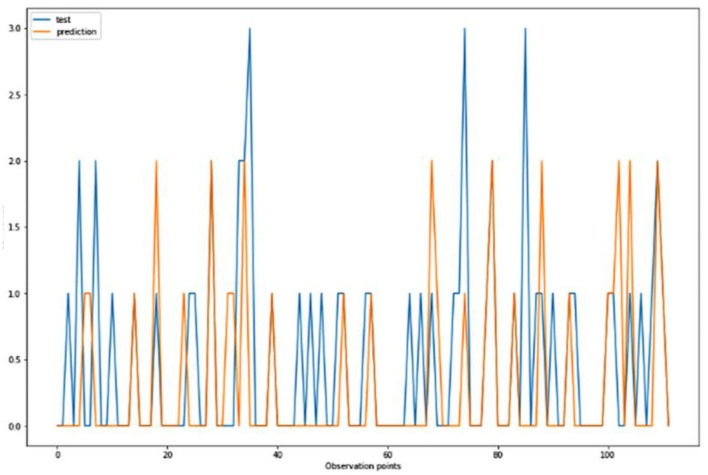
Graphical representation of the comparison of test and predicted CDR values obtained using the k-neighbors classification algorithm. (six parameters).

**Figure 6:**
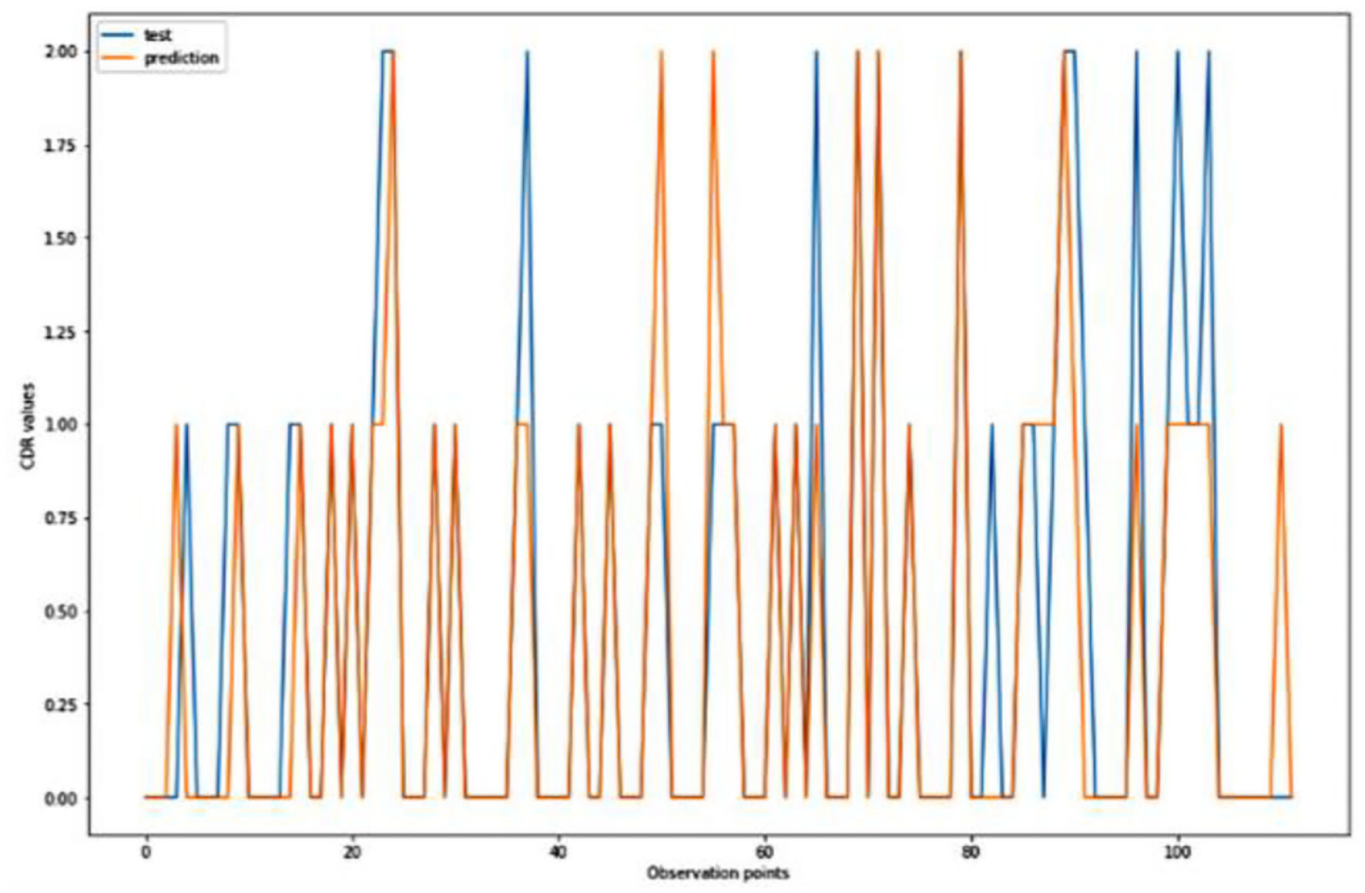
Graphical representation of the comparison of test and predicted CDR values obtained using the logistic algorithm. (11 parameters).

**Figure 7:**
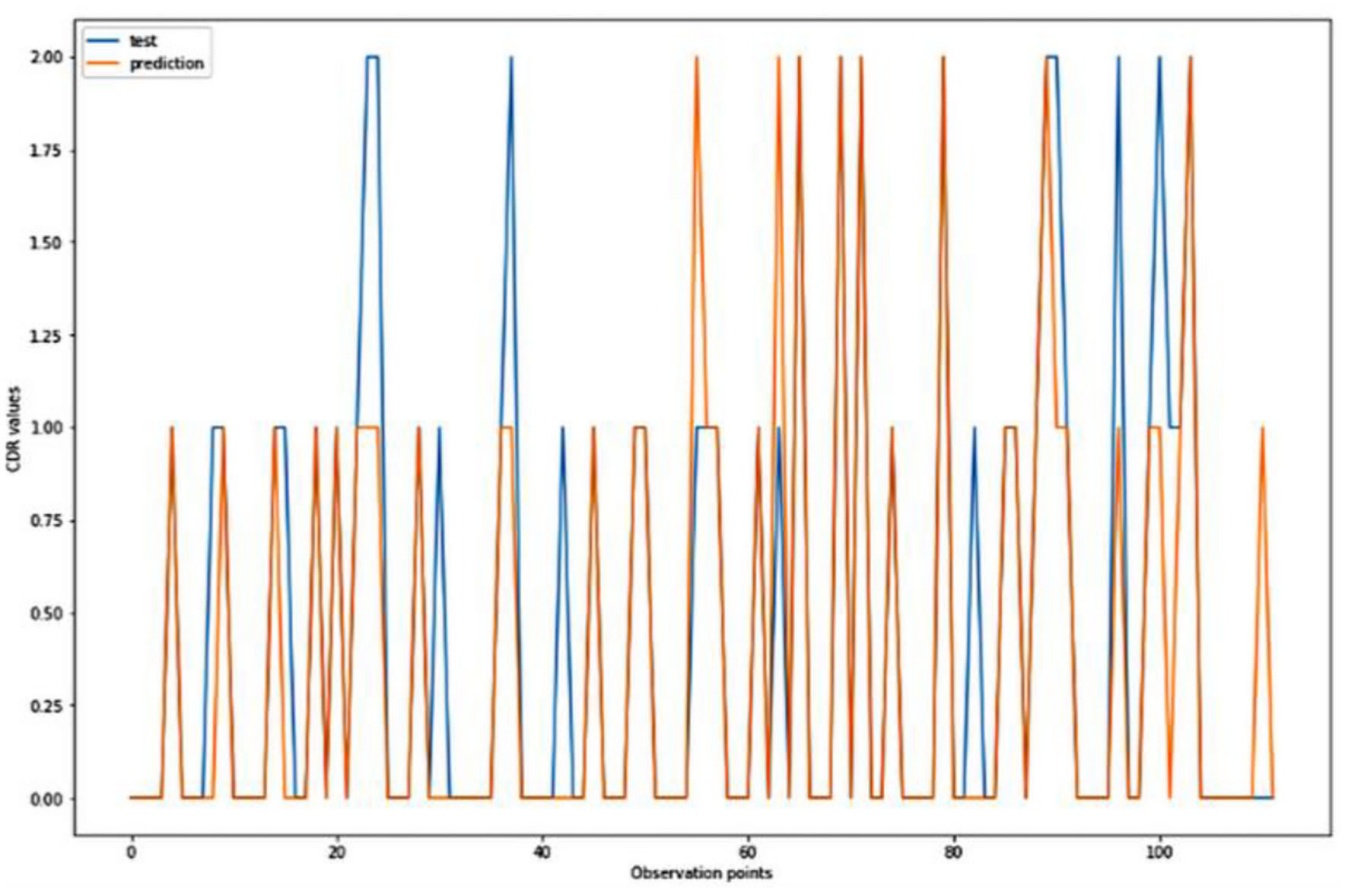
Graphical representation of the comparison of test and predicted CDR values obtained using the k-neighbors classification algorithm. (11 parameters).

**Figure 8:**
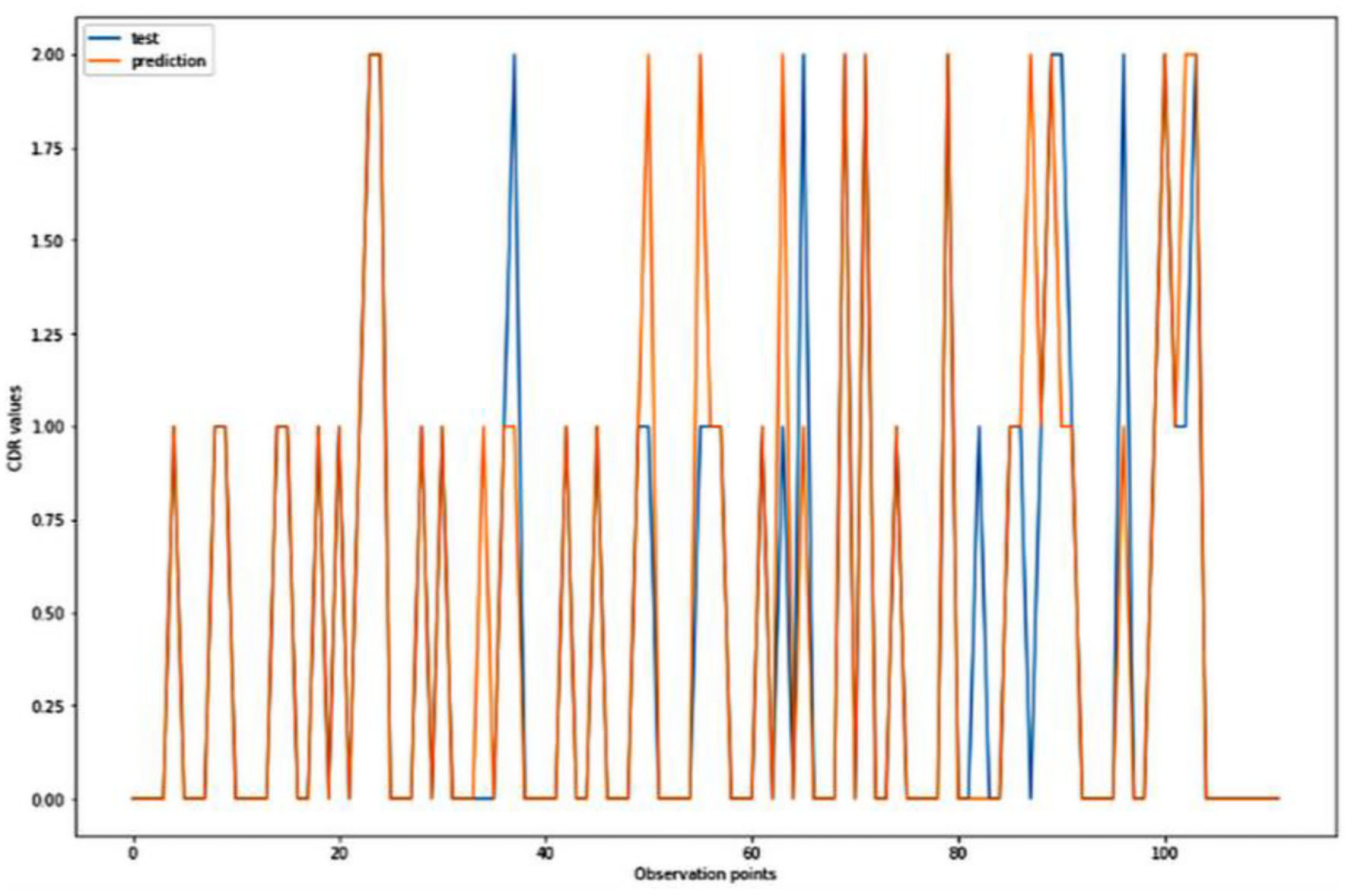
Graphical representation of the comparison of test and predicted CDR values obtained using the TensorFlow model. (11 parameters).

For the algorithm that predicts the onset (or worsening) of AD, the biomarkers and parameters were considered against the CDR score presented in Figure 4. The data was divided with a ratio of 7:3 and then fit into various classification models to predict the CDR scores. It is widely known that The CDR scores of 0 means “Normal,” 0.5 means “Very Mild Dementia,” 1 means “Mild Dementia,” 2 means “Moderate Dementia” and 3 means “Severe Dementia”. Accordingly, the results in the Figures 4–8 are interpreted for test and prediction. Further interpretations can be obtained from the table (36).

Figures 4–8 presented the CDR using the different parameters. Here, “sklearn” was used as a metric, whereas “TensorFlow” was used as a deep neural network classifier. There are two sets of results obtained—one by training the algorithm with the longitudinal and cross-sectional datasets, the other because of concatenating the datasets. The results obtained on training the neural network with the cross sectional and longitudinal datasets varied extensively for different models of classification. Here, results refer to the percentage of accuracy with which the model predicts onset of dementia or AD. The datasets were split into training data and test data using the CDR model with the training data: test data ration equal to 7:3. For the longitudinal dataset, the training data successfully predicted the onset of Alzheimer's with an accuracy of 83–88% for different classification models such as K Nearest Neighbors, SVM, TensorFlow, Standard Scalar and Logistic Regression, whereas the test data predicted the same result with an accuracy of 60–70%. The graphical representation of the correlation matrix proved that the dataset is random in nature and does not have any hidden pattern in it. On the other hand, when the datasets were classified using the CDR and MMSE scores and comparing them with the MRI scans, such that a ratio of 7:3 is maintained between the training data and test data set populations, the training data, and the test data both predicted the onset of AD with an accuracy of ~85%.

**Figure 9:**
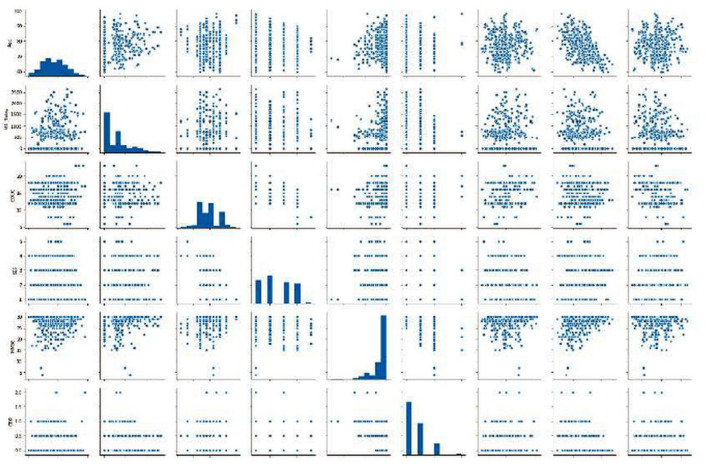
Graphics of scatter plots representing the correlation matrix of individual parameters against the rest of the parameters in consideration.

Different scatter plots (Figure 9) are also plotted which represent the correlation matrix of individual parameters against the rest of the parameters in consideration. Different scatter plots (Figure 9) are also plotted which represent the correlation matrix of individual parameters against the rest of the parameters in consideration. Summarized results are presented in Tables 1, 2.

**Table 1:** Accuracy obtained to predict AD in 2 years on the training and test sets.

**Models**	**Accuracy**			
	**six Parameters (M/F, Age, EDUC, SES, eTIV, ASF)**		**11 Parameters (M/F, Age, EDUC, SES, eTIV, ASF**, **Group, Hand, MMSE, MR Delay, nWBV)**	
	**Training Set**	**Test Set**	**Training Set**	**Test Set**
Logistic Regression	59.00%	67.86%	83.14%	78.57%
Decision Tree Classifier	98.47%	72.32%	95.40%	86.61%
K Nearest Neighbor	86.21%	68.75%	90.42%	79.46%
Support Vector Classifier	52.87%	60.71%	–	–

**Table 2:** Prediction accuracy for AD.

**Models**	**Prediction Accuracy**	
	**six Parameters (M/F, Age, EDUC, SES, eTIV, ASF)**	**11 Parameters (M/F, Age, EDUC, SES, eTIV, ASF, Group, Hand, MMSE, MR Delay, nWBV)**
Sklearn	70.54%	84.82%
TensorFlow	70.54%	84.82%

### Preventive Model

We further implemented a system which would display the preventive measures that should be taken by a patient (patient ID is provided as input) to prevent onset or control the severity of AD. The measures given for a particular patient were according to the present condition of the patient and keeping in mind that the condition will worsen (since AD is degenerative) over time if preventive measures are not taken. Any medication, if and wherever suggested were in accordance with the U.S. Food and Drug Administration (FDA). Also, medications were only suggested for the patients who are currently suffering from AD.

## Result Analysis

The predictive and preventive models were implemented successfully. The predictive model consists of two parts: to predict This model can further be improved upon by predicting the individuals who could develop AD or whose condition can worsen, instead of predicting for the whole dataset. Moreover, including genetic mappings, psychometric tests, mini mental state examination tests along with hand drawn images and shapes, etc. can make this whole system more comprehensive for the prediction of Alzheimer's disease. Systems with higher specifications can reduce the execution time. The algorithm can be further optimized and generalized using fuzzy logic concepts, such as fuzzy c-means instead of k-means algorithm which is essentially binary logic concept as implemented in this algorithm. Furthermore, hybrid models for clustering algorithms can also be used. One of the important prospects of this algorithm is to identify whether the preventive measures influence the subjects, either with mild, early stage or severe AD and to analyze the same based on MMSE and CDR scores over a period. Finally, the model can also be tested for scalability on big data (25, 30, 36–38). Whether a person has AD or not, using the T1 weighted coronal brain scan MRI, and to predict the severity of AD in the next 2 years, using the data from processed MRI images and through amyloid protein concentration. This achieved an accuracy of almost 85% and can be further improved as the number of executions is increased and data is added.

## Conclusions

The preventive model was based on the present condition of the patient and the predictive model. The preventive measures for each patient can be obtained by giving the ID of the patient as an input. With the current treatments of AD only tackling the symptoms, research is needed to check the onset of AD along with a way to deal with the biological changes that are responsible for it. With more data on the patients of AD (or suspected) the algorithms can be improved upon. As is evident, more parameters improve the accuracy of the algorithm hence; research on more biomarkers that play a role in the progression of AD is required.

## Data Availability Statement

Data were provided by OASIS Cross-Sectional and Longitudinal: Principal Investigators: D. Marcus, R, Buckner, J, Csernansky J. Morris; P50 AG05681, P01 AG03991, P01 AG026276, R01 AG021910, P20 MH071616, and U24 RR021382.

## Author Contributions

All authors listed have made a substantial, direct and intellectual contribution to the work and approved it for publication.

## Conflict of Interest

The authors declare that the research was conducted in the absence of any commercial or financial relationships that could be construed as a potential conflict of interest.

## Publisher's Note

All claims expressed in this article are solely those of the authors and do not necessarily represent those of their affiliated organizations, or those of the publisher, the editors and the reviewers. Any product that may be evaluated in this article, or claim that may be made by its manufacturer, is not guaranteed or endorsed by the publisher.
